# Validation of the Diet Satisfaction Questionnaire: a new measure of satisfaction with diets for weight management

**DOI:** 10.1002/osp4.299

**Published:** 2018-10-10

**Authors:** B. L. James, E. Loken, L. S. Roe, K. Myrissa, C. L. Lawton, L. Dye, B. J. Rolls

**Affiliations:** ^1^ Department of Nutritional Sciences The Pennsylvania State University, University Park PA USA; ^2^ Department of Human Development and Family Studies The Pennsylvania State University, University Park PA USA; ^3^ Human Appetite Research Unit (Nutrition and Behaviour Research Group), School of Psychology University of Leeds Leeds UK

**Keywords:** Adults, diet, obesity treatment, weight loss

## Abstract

**Objective:**

The Diet Satisfaction Questionnaire was developed to fill the need for a validated measure to evaluate satisfaction with weight‐management diets. This paper further develops the questionnaire, examining the factor structure of the original questionnaire, cross‐validating a revised version in a second sample and relating diet satisfaction to weight loss during a 1‐year trial.

**Methods:**

The 45‐item Diet Satisfaction Questionnaire (DSat‐45) uses seven scales to assess characteristics that influence diet satisfaction: Healthy Lifestyle, Convenience, Cost, Family Dynamics, Preoccupation with Food, Negative Aspects, and Planning and Preparation. It was administered five times during a 1‐year weight‐loss trial (*n* = 186 women) and once as an online survey in a separate sample (*n* = 510 adults). Confirmatory factor analysis was used to assess and refine the DSat‐45 structure, and reliability and validity data were examined in both samples for the revised questionnaire, the DSat‐28. Associations were examined between both DSat questionnaires and weight loss in the trial.

**Results:**

Internal consistency (reliability) was moderate for the DSat‐45. Confirmatory factor analysis showed improved fit for a five‐factor structure, resulting in the DSat‐28 that retained four of the original scales and a shortened fifth scale. This revised questionnaire was reliable in both samples. Weight loss across the year‐long trial was positively related to satisfaction with Healthy Lifestyle, Preoccupation with Food, and Planning and Preparation in both versions of the questionnaire.

**Conclusions:**

Measures of reliability and validity were improved in the more concise DSat‐28 compared to the DSat‐45. This shorter measure should be used in future work to evaluate satisfaction with weight‐management diets.

## Introduction

There is a need in the weight‐management field for a measure of satisfaction with an individual's current diet. In behavioural weight‐loss interventions, the degree of adoption and maintenance of the prescribed diet is a strong predictor of the magnitude of long‐term weight loss 1, 2. Weight‐loss outcomes vary widely across individuals, and although this occurs for multiple reasons, an important cause is lack of adherence to a diet that promotes energy restriction 3. Often, little is known about how the recommended diet is perceived by those receiving intervention, but dissatisfaction with the effect of the diet on daily life may make it difficult to adopt and sustain the prescribed dietary modifications 4. Therefore, understanding the characteristics of a dietary program that facilitate or hinder weight management is critical. One possible instrument for this use, the 45‐item Diet Satisfaction Questionnaire (DSat‐45) 5, measures key aspects such as diet cost and convenience. This paper extends the initial validation of the DSat‐45 6, 7, 8 by assessing its factor structure in order to offer a refined version of the questionnaire for future use and by examining the relationship between diet satisfaction and weight loss during a 1‐year trial.

Questionnaires concerning satisfaction in weight‐loss trials commonly measure general satisfaction or quality of life without specific questions about the diet itself. For example, validated questionnaires have shown the impact of obesity on quality of life as well as the improvements that result from weight loss 9, 10, 11. Additionally, satisfaction with the type of intervention 12, 13 and with the initial amount of weight loss 14 have been shown to predict long‐term weight loss. These broad measures would be supported by administering a more specific questionnaire related to diet satisfaction, such as the DSat‐45.

We examined the factor structure of the DSat‐45 and the associations with weight loss using multiple datasets and larger samples than previously assessed, in order to strengthen preliminary findings and improve the questionnaire. In addition, the DSat‐45 has not previously been administered repeatedly in a longer‐term trial; such multiple assessments over time would strengthen the conclusions that can be made about the reliability and validity of the measure. Therefore, the purpose of our analyses was to assess the 45‐item Diet Satisfaction Questionnaire longitudinally, to evaluate the reliability and validity of the DSat‐45 and any proposed revision in two separate samples (a 1‐year weight‐loss trial and a one‐time online survey administration), and to offer recommendations for its future use.

## Methods

### Questionnaire

The Diet Satisfaction Questionnaire was designed to provide specific assessment of satisfaction as it relates to following a weight‐management diet. The questionnaire was originally developed and tested in 97 women participating in a weight‐loss trial 5. During that trial, questions about diet satisfaction were pilot tested, and the number of questions was reduced using principal components analysis to eliminate those with poor fit. This process of initial validation yielded a 45‐item questionnaire measuring characteristics of the lifestyle and attitudes of individuals that reflected satisfaction with their current diets. Using principal components analysis 5, the 45 statements were grouped into seven scales of diet satisfaction: Healthy Lifestyle, Convenience, Cost, Family Dynamics, Preoccupation with Food, Negative Aspects, and Planning and Preparation (Table 1). The items are assessed using five responses ranging from ‘Disagree Strongly’ to ‘Agree Strongly’, which are scored from 1 to 5. Items are reverse‐scored if necessary, so that higher scores indicate greater diet satisfaction, and scale scores are created by averaging scores across items. A Total Diet Satisfaction score is also calculated by averaging all item scores. Table 2 provides the wording of the items for each scale in the DSat‐45.

**Table 1:** Scale structure of the original version of the Diet Satisfaction Questionnaire (DSat‐45)

Scale	Scale description	Number of items
Healthy Lifestyle	Degree to which the diet supports a Healthy Lifestyle and promotes positive feelings about life	8
Convenience	Ease of finding foods that fit within the diet at restaurants and grocery stores	9
Cost	Financial cost of the diet	5
Family Dynamics	Family support of, and attitudes toward, the individual following the diet	6
Preoccupation with Food	Tendency to think about food and hunger between meals	6
Negative Aspects	Negative feelings of following the diet, such as deprivation, self‐consciousness or inconvenience	6
Planning and Preparation	Amount of time and effort spent in planning and preparing food on the diet	5

**Table 2:** Original 45 items of the seven‐scale Diet Satisfaction Questionnaire (DSat‐45©), with revised items and scales of the five‐scale version (DSat‐28©) indicated in bold

Scale	Original	Item
	Item #	Wording
**Healthy**	1	***I have a lot of energy*.**
**Lifestyle**	2	***I feel good about myself*.**
	3	***I think that I eat a healthy diet*.**
	4	***I believe that I am reducing my risk for disease by the way that I eat*.**
	5	***I believe that I am reducing my risk for disease by the way that I exercise*.**
	6	***I think that I have a healthy lifestyle*.**
	7	***I am satisfied with my current diet*.**
	8*	***The way that I currently eat makes me feel guilty*.**
Convenience	9*	***The way I currently eat prevents me from eating in restaurants frequently*.**
**[Eating Out in**	10	***When dining out, I can easily choose foods from the menu that fit into my current diet*.**
**DSat‐28]**	11*	***Finding appropriate food choices at restaurants is difficult*.**
	12*	*I have to prepare most of my foods ‘from scratch’*.
	13	*I find eating satisfying*
	14*	***I have difficulty finding the foods I want when eating out*.**
	15	*I find it easy to shop at my grocery store for the kinds of foods I eat*.
	16*	*I limit my choice of restaurants*.
	17	*I have plenty of different types of foods to choose from with my current diet*.
**Cost**	18*	***I feel that I spend a large amount of my budget on the foods that I eat*.**
	19	***I think that preparing food and meals for the way I eat now is economical*.**
	20*	***I think that preparing food and meals for the way I eat now costs a lot of money*.**
	21*	***I spend a lot of money on food*.**
	22*	***It is hard for me to afford the kind of foods that I eat*.**
Family	23*	*I feel that the way I eat now bothers my family*.
Dynamics	24	*My family encourages me to keep eating the way I am eating now*
	25	*My family supports my efforts to eat a healthy diet*.
	26	*I enjoy getting together for holiday meals with family*.
	27*	*My family discourages me from eating the way I am eating now*.
	28*	*The way I currently eat causes stress within my family*.
**Preoccupation**	29*	***Thoughts of food are always on my mind*.**
**with Food**	30*	***I think about food between almost every meal*.**
	31*	***I have cravings for some of my favourite foods*.**
	32*	***I always feel like I want to snack between meals*.**
	33*	***I often feel hungry*.**
	34*	***I feel that my diet controls my life*.**
Negative	35*	*I feel deprived based on what I order when eating in a restaurant*.
Aspects	36*	*I feel self‐conscious trying to eat my current diet at social events*.
	37*	*I feel embarrassed if I order specially prepared foods in a restaurant*.
	38	*My family eats the same foods that I currently eat*.
	39*	*I feel deprived when I choose to avoid some of my favourite foods*.
	40*	*I have to prepare separate meals for my family and myself*.
**Planning and**	41*	***I spend a lot of time planning my meals*.**
**Preparation**	42*	***I spend a lot of time shopping for food*.**
	43*	***I think preparing foods and meals for the way I eat now is time‐consuming*.**
	44*	***I think preparing food and meals for the way I eat now requires a lot of effort*.**
	45*	***I spend a lot of time looking for new ideas for food and meals that fit into my current diet*.**

Scales are measured using five responses ranging from ‘Disagree Strongly’ to ‘Agree Strongly’, which are scored from 1 to 5. Items are reverse‐scored if necessary (indicated by an asterisk), so that higher scores indicate greater diet satisfaction. Item scores are averaged to provide scale scores.

The scales represent a broad range of constructs. For instance, the Healthy Lifestyle scale represents satisfaction with overall physical health, particularly the diet's contribution, which appears to be important for weight loss. The Planning and Preparation measures time, thought and effort spent on the diet. The Preoccupation with Food scale reflects the extent that food‐centered thoughts, potentially reduced through greater satiety, relates to weight loss.

The DSat‐45 has been administered several times in different populations. Analysis of the questionnaire in the original weight‐loss trial 5 found that compared to baseline diets, both intervention diets significantly improved satisfaction with supporting a healthier lifestyle, having fewer negative aspects, and leading to less preoccupation with food 6. Findings from another group showed increases in diet satisfaction during a dietary intervention and correlations with attendance and compliance 7. The only other study examining the factor structure of the DSat‐45, however, found that a six‐factor alternative offered a stronger fit and showed mixed results for the internal consistency of some factors 8, suggesting a need for further refinement of the questionnaire. The aim of the present analyses was to evaluate the reliability and validity of the DSat‐45 in two separate samples and to identify any improvements in the questionnaire for the purpose of evaluating diet satisfaction in future studies.

### Participants and design: Sample 1

Sample 1 consisted of 186 women with overweight or obesity from central Pennsylvania who were enrolled in a 1‐year randomized controlled trial examining the effect of portion‐control strategies on weight management. The women had a mean (±SD) age of 50.0 ± 10.6 years and a mean body mass index (BMI) of 34.0 ± 4.2 kg/m^2^. Details of the Portion‐Control Strategies Trial design and outcomes are published elsewhere 15. In brief, participants were randomly assigned to one of three groups. The Standard Advice Group was taught to follow the Dietary Guidelines 16 to eat less and make healthy choices from all food groups. The Pre‐portioned Foods Group received vouchers for pre‐portioned meals and was taught to use other pre‐portioned foods to manage intake. The Portion Selection Group was given tools such as food scales and taught strategies such as using energy density to select portions. The trial protocol was approved by the Office for Research Protections at The Pennsylvania State University.

All participants received an equal amount of individual time with trained interventionists, consisting of 19 educational sessions and five assessment sessions over the course of 1 year. Body weight was measured at each session, and the Diet Satisfaction Questionnaire (DSat‐45) was completed at each of the five assessment sessions: baseline and Months 1, 3, 6 and 12. Questionnaire completion rates were 100% at baseline and Month 1, 94% at Month 3, 83% at Month 6 and 76% at Month 12. The main finding of the Portion‐Control Strategies Trial 15 was that there were significant differences across intervention groups in the trajectories of weight loss over the year. In the initial months of intervention, the Pre‐portioned Foods Group lost weight at a faster rate than the other two groups, and during later months they regained weight at a faster rate than the other groups. There were no significant differences in mean (±SEM) weight loss across groups at Month 6 (5.2 ± 0.4 kg) or Month 12 (4.5 ± 0.5 kg).

### Participants and design: Sample 2

Sample 2 consisted of 510 adults from the United Kingdom who participated in a one‐time online survey developed at the University of Leeds. The DSat‐45 was administered in this sample as part of a series of questionnaires to identify psychological and behavioural characteristics of adults who had previously attempted weight loss using behavioural strategies. Participants were primarily female (73%) and in the 18–34 year age range (77%). The survey was advertised using social media and on posters placed in and around Leeds. Participants were eligible if they had attempted to lose weight in the last 6 months, were age 18–65 years and were not currently pregnant or breastfeeding. Height and weight were self‐reported and only 38% complete, and are therefore not evaluated in these analyses. The DSat‐45 was completed once by each respondent, and there were no missing responses on the questionnaire. The study was approved by the Institute of Psychological Sciences Ethics Committee of the University of Leeds.

### Statistical analyses

Data from both Sample 1 and Sample 2 were used to assess the reliability (internal consistency) and validity of the DSat‐45 in order to determine its adequacy and whether to revise its content. Internal consistency among the items of each scale was evaluated by Cronbach's alpha, and correlation between scales was assessed by Pearson correlation coefficients. Confirmatory factor analysis was conducted on Sample 1 data (pooled across all five time points) to determine the fit of the seven‐scale DSat‐45 structure. Standard criteria were used to evaluate several goodness‐of‐fit indices: standardized root mean square residual (SRMR), root mean square error of approximation (RMSEA), comparative fit index (CFI) and normed fit index (NFI) 17, 18.

Revisions to the questionnaire were based on the results from the Sample 1 factor analysis and guided by previously defined methods 19, summarized as follows. Scales with multiple items that loaded poorly (< 0.30) or cross‐loaded were considered for refinement or removal. Individual items were also considered for removal if they loaded poorly and if the remaining scale items reflected a clearer theoretical construct. Poorly performing scales were removed from the model before considering individual items for removal; item wording was not revised in this process. Modification indices were used to guide the scale revision process and to improve model fit, but the factor structure indicated by the confirmatory factor analysis results was the primary determinant of any revisions. Internal consistency and correlation between scales were again assessed for the refined questionnaire structure. Finally, confirmatory factor analysis was conducted with Sample 2 data to evaluate the fit of the reduced measure and to use Sample 2 data to cross‐validate the findings from Sample 1. These analyses enabled assessment of the consistency of the findings in a different sample and context.

Changes in diet satisfaction ratings during intervention in the weight‐loss trial were assessed for descriptive purposes. A linear mixed model with repeated measures was used to evaluate changes in the scale scores of both the DSat‐45, and the revised questionnaire across the five assessment time points in Sample 1. The Tukey–Kramer method was used to adjust for multiple comparisons between mean scores.

In order to assess the relationship of the questionnaire to a clinically relevant outcome, the influence of the DSat scale scores on the trajectory of weight loss across all time points of the trial was analysed with random coefficients models, using maximum likelihood methods to handle missing data. In an intention‐to‐treat analysis, individual trajectories of weight loss were modelled for all randomized subjects using the available data. Linear and quadratic effects of time (trial week) were included as fixed factors, and all models were controlled for intervention group as well as baseline BMI and age. Each DSat scale score was included separately as a covariate, first as a baseline value only and then as a time‐varying covariate controlling for the baseline value, to determine the individual relationship of each scale with the weight‐loss trajectory. The model was then run with all DSat scales included as covariates to determine the relative strength of the relationships with weight loss. These analyses were run on Sample 1 data for the DSat‐45 and for the revised version of the questionnaire that resulted from the factor analytic work. The data were analysed using SAS software (version 9.4, 2013, SAS Institute Inc., Cary, NC). Outcomes from mixed models are reported as mean ± SEM and were considered significant at *P* < 0.05.

## Results

### Questionnaire reliability and revision

#### DSat‐45: Reliability (internal consistency)

In Sample 1, all seven DSat‐45 scales showed acceptable internal consistency at each of the five assessment time points. Cronbach's alpha levels ranged from 0.68 for the Negative Aspects scale to 0.91 for the Healthy Lifestyle scale. Data in Sample 2 revealed similar patterns of internal consistency, with alpha levels ranging from 0.65 for the Convenience scale to 0.89 for the Healthy Lifestyle scale.

#### DSat‐45: Comparison of scale scores across time and samples

Table 3 shows the mean scale scores over time for participants in Sample 1, and mean scores for the single administration in Sample 2. In Sample 1, satisfaction ratings for Healthy Lifestyle, Preoccupation with Food, Family Dynamics and Total Diet Satisfaction showed a significant initial increase from baseline and remained elevated throughout the trial. Scale scores in Sample 2 were comparable to those in Sample 1 (Table 3).

**Table 3:** Mean scores^1^ (±SD) on the scales of the original (DSat‐45) and revised (DSat‐28) versions of the Diet Satisfaction Questionnaire across five time points for Sample 1 and for the single administration in Sample 2

	Sample 1 (*n* = 186)					Sample 2 (*n* = 510)
Scale	Baseline (*n* = 186)	Month 1 (*n* = 186)	Month 3 (*n* = 175)	Month 6 (*n* = 154)	Month 12 (*n* = 142)	(*n* = 510)
Healthy Lifestyle	2.60 ± 0.74^a^	3.86 ± 0.65^b^	3.72 ± 0.77^c^	3.81 ± 0.70^b^^,^ c	3.72 ± 0.76^c^	3.52 ± 0.88
Convenience^2^	3.76 ± 0.57^a^^,^ d	3.58 ± 0.52^b^	3.66 ± 0.56^a^^,^ b ^,^ c	3.74 ± 0.52^a^^,^ c ^,^ d	3.80 ± 0.53^d^	3.39 ± 0.54
Eating Out (DSat‐28)	3.70 ± 0.78^a^^,^ c	3.46 ± 0.82^b^	3.62 ± 0.84^a^^,^ b	3.81 ± 0.80^c^	3.87 ± 0.76^c^	3.33 ± 0.53
Cost	3.01 ± 0.75^a^	3.28 ± 0.72^b^	3.21 ± 0.71^b^	3.21 ± 0.73^a^^,^ b	3.14 ± 0.79^a^^,^ b	3.21 ± 0.74
Family Dynamics^2^	3.84 ± 0.66^a^	4.30 ± 0.66^b^	4.20 ± 0.70^b^	4.28 ± 0.67^b^	4.24 ± 0.65^b^	3.84 ± 0.67
Preoccupation with Food	2.78 ± 0.77^a^	3.11 ± 0.77^b^	3.07 ± 0.80^b^	3.14 ± 0.88^b^	3.16 ± 0.87^b^	2.95 ± 0.83
Negative Aspects^2^	3.69 ± 0.62^a^^,^ c ^,^ d	3.53 ± 0.73^b^	3.62 ± 0.69^a^^,^ b	3.80 ± 0.64^d^	3.88 ± 0.69^c^^,^ d	3.40 ± 0.69
Planning and Preparation	3.43 ± 0.80^a^	3.46 ± 0.77^a^	3.37 ± 0.77^a^	3.38 ± 0.82^a^	3.41 ± 0.82^a^	3.21 ± 0.79
Total Diet Satisfaction (DSat‐45)^2^	3.30 ± 0.37^a^	3.61 ± 0.41^b^	3.58 ± 0.44^b^	3.65 ± 0.44^b^	3.65 ± 0.44^b^	3.38 ± 0.52
Total Diet Satisfaction (DSat‐28)	2.83 ± 0.38^a^	3.05 ± 0.33^b^	3.03 ± 0.38^b^^,^ c	3.04 ± 0.37^b^^,^ c	2.99 ± 0.35^c^	3.05 ± 0.48

1 Scale scores range from 1 to 5, with higher scores indicating greater satisfaction.

2 This scale was omitted or revised in the DSat‐28.

a ^,^

b ^,^

c ^,^

d Means for the same scale with different letters are significantly different (*p* < 0.05).

#### DSat‐45: Confirmatory factor analysis

Confirmatory factor analysis of the DSat‐45 using the repeated measurements from Sample 1 found weak fit indices (SRMR: 0.09, RMSEA: 0.07, CFI: 0.78, NFI: 0.74) and multiple items that failed to load on any factor. In particular, two scales (Family Dynamics and Negative Aspects) had poor internal consistency and multiple items that loaded poorly. Although the Family Dynamics scale addressed a topic of interest and showed changes during intervention, this scale was eliminated due to poor fit. The Negative Aspects scale showed inconsistent item loading and no significant changes during intervention. On a third scale (Convenience), only four of the nine items loaded together. Items on the remaining four DSat‐45 scales showed acceptable factor loading and internal consistency.

#### DSat‐28: Revision and confirmatory factor analysis

The factor analysis of the DSat‐45 in Sample 1 led to the removal of the scales with poor internal consistency (Family Dynamics and Negative Aspects). The Convenience scale was truncated to the four items that loaded together, and based on their content this scale was renamed the Eating Out scale. This process resulted in a revised 28‐item questionnaire with five scales (DSat‐28). Table 2 identifies the revised scales and items that were retained in the new version of the questionnaire; this version was not independently administered in either sample.

Confirmatory factor analysis of the DSat‐28 questionnaire in Sample 1 showed that fit indices for the revised structure were improved (SRMR: 0.07, RMSEA: 0.07, CFI: 0.86, NFI: 0.84). Table 4 shows the factor loadings for the DSat‐28 using the data from Sample 1. The revised questionnaire was cross‐validated in Sample 2, yielding a similar pattern of fit indices as found in Sample 1 (SRMR: 0.08, RMSEA: 0.08, CFI: 0.86, NFI: 0.83). Thus, the cross‐validation in a different sample showed that the revised version of the questionnaire performed comparably across different populations and contexts.

**Table 4:** Confirmatory factor analysis of the revised Diet Satisfaction Questionnaire (DSat‐28) using data from a 1‐year weight‐loss trial (Sample 1; *n* = 186)

Scale	Revised item #	Factor 1	Factor 2	Factor 3	Factor 4	Factor 5
Healthy Lifestyle	1	0.60				
	2	0.65				
	3	0.86				
	4	0.87				
	5	0.70				
	6	0.83				
	7	0.81				
	8	0.63				
Eating Out	9		0.48			
	10		0.59			
	11		0.82			
	12		0.76			
Cost	13			0.74		
	14			0.49		
	15			0.79		
	16			0.84		
	17			0.54		
Preoccupation with Food	18				0.85	
	19				0.87	
	20				0.43	
	21				0.54	
	22				0.61	
	23				0.64	
Planning and Preparation	24					0.51
	25					0.59
	26					0.90
	27					0.87
	28					0.44

All values are standardized regression weights representing factor loadings

#### DSat‐28: Internal consistency and reliability of the new questionnaire

The internal consistency measures for the DSat‐28 questionnaire are shown in Table 5 for the two samples. In Sample 1, the revised structure showed overall improvement in internal consistency, ranging from 0.75 for the Eating Out scale to 0.91 for the Healthy Lifestyle scale. A similar pattern was seen for the DSat‐28 in Sample 2, with alpha levels ranging from 0.73 to for the Eating Out scale to 0.89 for the Healthy Lifestyle scale, indicating a consistent, improved fit for the revised structure. The same patterns of correlation between scales of the DSat‐28 were observed in both samples (Table 5), including between Cost and Preoccupation with Food, Cost and Planning and Preparation, and Preoccupation with Food and Healthy Lifestyle.

**Table 5:** Internal consistency within and correlations between scales for the revised, five‐scale Diet Satisfaction Questionnaire (DSat‐28)

Sample 1 (*n* = 186)	Cronbach's alpha	Pearson correlation coefficient^1^				
		Healthy Lifestyle	Eating Out	Cost	Preoccupation with Food	Planning and Preparation
Healthy Lifestyle	0.91	1.00				
Eating Out	0.75	0.07	1.00			
Cost	0.81	0.21*	0.17*	1.00		
Preoccupation with Food	0.83	0.39*	0.23*	0.24*	1.00	
Planning and Preparation	0.81	0.07	0.19*	0.39*	0.34*	1.00
Sample 2 (*n* = 510)	Cronbach's alpha	Pearson correlation coefficient^1^				
		Healthy Lifestyle	Eating Out	Cost	Preoccupation with Food	Planning and Preparation
Healthy Lifestyle	0.89	1.00				
Eating Out	0.73	0.05	1.00			
Cost	0.82	0.20*	0.19*	1.00		
Preoccupation with Food	0.87	0.33*	0.30*	0.33*	1.00	
Planning and Preparation	0.85	0.06	0.29*	0.43*	0.34*	1.00

* Correlation is significantly different from 0 (*p* < 0.001).

1 Correlations were calculated across five time points for Sample 1 and for the single administration for Sample 2.

Validation and intervention effects.

#### DSat‐45: Relationships with weight across time

The baseline scores for the original DSat‐45 scales, which reflected the pre‐intervention diet, were not related to the subsequent trajectory of weight loss over time. During intervention, however, three of the seven DSat‐45 scales were related to the rate of weight loss across time when examined as a time‐varying covariate. The scales for Healthy Lifestyle (*P* < 0.0001), Preoccupation with Food (*P* < 0.0001), and Planning and Preparation (*P* = 0.02), as well as the score for Total Diet Satisfaction (*P* < 0.0001), were positively related to weight loss over the 12 months of the trial. Higher scores on these scales, indicating greater satisfaction with the diet, were associated with a greater magnitude of weight loss. The remaining four scales of the DSat‐45 did not show any relationship with weight loss (all *P* > 0.40).

When the seven scales of the DSat‐45 were included in the same model, the scales for Healthy Lifestyle (*P* < 0.001), Preoccupation with Food (*P* = 0.01), and Planning and Preparation (*P* = 0.07) remained significantly related to weight change or trended towards significance.

#### DSat‐28: Relationship with weight across time

In developing the revised DSat‐28 based on factor analysis, the three scales of the DSat‐45 that were found to relate to weight loss across time were not altered, nor was the wording changed for any items. Furthermore, the scales that were removed from the DSat‐45 to create the DSat‐28 did not show any relationship with weight change. Although the DSat‐28 was not administered to the sample populations, parallel analyses were conducted to determine the associations with weight loss when only these 28 items were included. Similar to the findings reported above, the scales for Healthy Lifestyle (*P* < 0.0001), Preoccupation with Food (*P* < 0.0001), Planning and Preparation (*P* = 0.02), and Total Diet Satisfaction (*P* < 0.0001) were positively related to weight loss over the 12 months of the trial. These relationships are indicated by the fixed effects coefficients in Table 6, which represent the magnitude of change in weight loss (kg) per unit change in score for each DSat‐28 scale. As an example, participants in the highest tertile of Healthy Lifestyle score lost 6.1 ± 0.4 kg after a year of intervention, compared to those in the lowest tertile who lost 2.4 ± 0.2 kg. A similar pattern was found for the scales of Preoccupation with Food (highest tertile: 5.7 ± 0.4 kg; lowest tertile: 3.4 ± 0.3 kg) and Planning and Preparation (highest tertile: 5.3 ± 0.4 kg; lowest tertile: 3.5 ± 0.3 kg). Results from a combined analysis of the five DSat‐28 scales identified significant or marginally significant relationships with weight loss for the scales of Healthy Lifestyle (*P* < 0.001), Eating Out (*P* = 0.04) and Preoccupation with Food (*P* = 0.05). Together, these findings offer further validity for the revised DSat‐28 questionnaire.

**Table 6:** Results of random coefficients models examining the relationships between scales of the Diet Satisfaction Questionnaire (DSat‐28) and the trajectory of weight loss (kg) during a 1‐year trial (Sample 1; *n* = 186 women)

Variable	Base model coefficient (mean ± SEM)	Significance	Fixed effect coefficient (mean ± SEM)	Significance
**Fixed effects included in all models**				
Time, linear (week)	0.57 ± 0.05	*P* < 0.001		
Time, quadratic (week*week)	−0.01 ± 0.000	*P* < 0.001		
Baseline BMI (kg/m^2^)	0.005 ± 0.006	*P* = 0.42		
Baseline age (years)	0.000 ± 0.000	*P* = 0.15		
**Fixed effects tested individually** 1				
Healthy Lifestyle scale			1.16 ± 0.11	*P* < 0.001
Eating Out scale			−0.09 ± 0.10	*P* = 0.37
Cost scale			0.10 ± 0.12	*P* = 0.40
Preoccupation with Food scale			0.75 ± 0.11	*P* < 0.001
Planning and Preparation scale			0.25 ± 0.10	*P* = 0.02
Total Diet Satisfaction			1.23 ± 0.22	*P* < 0.001

BMI: body mass index

1 Scales were included separately in the model to determine their individual relationship with weight loss. Results from models including all scales together are included in the text.

## Discussion

The 28‐item Diet Satisfaction Questionnaire provides a new, valid instrument for assessing diet satisfaction in the context of a weight‐management diet. Furthermore, satisfaction ratings were found to be related to the trajectory of weight loss over time in a controlled trial. Acceptable internal consistency and reliability were shown in both participant samples for the 45‐item, seven‐scale structure of the questionnaire (DSat‐45), but further analysis indicated that a 28‐item, five‐scale structure (DSat‐28) offered substantial improvements. The revisions made the questionnaire more concise and focused on the scales that showed strong reliability and validity, as well as relationships with weight loss across time. Results of the validation were found to be comparable in two large and varied samples using different study designs, thus supporting the use of the questionnaire in different contexts. The data reported here validate the 28‐item, five‐scale version of the questionnaire, which is recommended for future use in assessing ratings of satisfaction with different aspects of weight‐management diets.

Weight loss outcomes vary substantially across individuals, and the constructs measured by the DSat‐28 are likely to impact an individual's ability to adhere to a diet that promotes weight management 3, 4. In this study, satisfaction with how one's diet supports a Healthy Lifestyle was strongly related to weight change over time, which suggests that in developing weight‐loss interventions, participant perceptions of the quality of the prescribed diet require consideration. Relationships with the Planning and Preparation and Preoccupation with Food scales highlight the impact of thought and effort on overall diet satisfaction.

Although initial weight loss is achievable for many, it tends to plateau, followed by weight regain. Lack of adherence to the lifestyle changes that produced the weight loss, of which diet is often key, is a major contributor to this regain 3. With the ability to assess changes in satisfaction with the current diet, interventionists could develop strategies to help individuals re‐commit to a diet plan or introduce novelty in the diet in a way that promotes adherence. Using the DSat‐28 to assess satisfaction with and acceptance of a prescribed diet could contribute to our understanding of the variables that predict individual weight management. Low satisfaction would indicate the need to adjust intervention to better fit the individual, in order to improve long‐term adherence by eliminating barriers to adoption. Repeated administration during treatment could also identify changes in diet satisfaction, which might predict changes in adherence, which in turn likely affect weight loss.

The participants assessed in this study were predominantly female, limiting conclusions about different aspects of diet satisfaction in men. However, this study does show consistent factor structure for the questionnaire in different age ranges, since one study primarily assessed women over the age of 40, while the other evaluated men and women primarily under age 35. Dietary data were not collected for Sample 2 participants; this lack of information precluded investigating the effect of current dieting status on the outcome of diet satisfaction. However, the validation findings were consistent in both samples despite the large difference in settings (a single‐administration online survey in a free‐living European sample compared to repeated assessment in a weight‐loss trial in the US).

The revised Diet Satisfaction Questionnaire (DSat‐28) should have utility in weight‐loss treatment, more general dietary interventions and in non‐treatment contexts. Its ability to assess diet satisfaction both within and outside the context of weight‐loss treatment, as well as to assess change in satisfaction as a result of treatment, make it useful in a variety of settings. Reduced participant burden in the shortened DSat‐28 also facilitates use in such settings compared to the original DSat‐45. Future studies should broaden these findings by administering the DSat‐28 in additional populations and settings. The data from this study provide preliminary evidence for the validity of the revised version of the Diet Satisfaction Questionnaire, establishing the DSat‐28 as a valid measure of different aspects of satisfaction with weight‐management diets.

## Disclosures

BJR receives royalties from the sale of the Volumetrics books. BLJ, EL, LSR, KM, CLL and LD declare no conflict of interest.
